# How Autism Assistance Canines Enhance the Lives of Autistic Children

**DOI:** 10.1177/00469580231195029

**Published:** 2023-08-24

**Authors:** Sinéad Morgan, Dr Anne O’Byrne

**Affiliations:** 1 Mary Immaculate College, Co. Limerick, Ireland

**Keywords:** autism assistance canine, autism spectrum (AS), animal assisted education, child canine bond

## Abstract

The primary objective of this qualitative study was to examine the emerging phenomenon regarding the use of autism assistance canines to support the development of autistic children. Following an evaluation and analysis of literature and national educational policies, it became apparent that, to date, little research has been conducted regarding the concept of animal assisted education within Irish society. Therefore, this qualitative research study aimed to increase the body of research in order to inform future policy makers. Data were gathered using 9 semi structured interviews which explored the attitudes, experiences and perceptions of parents, canine handlers and teachers. Thematic analysis was used to assess, analyse, code and identify themes. The results clearly indicated that an autism assistance canine can positively enhance the life experience of an autistic child in Irish society. More specifically, this study found that an autism assistance canine can positively influence the behavior, safety, social interaction, independent functioning, companionship, language development, educational experience and the family life of an autistic child.

**What do we already know about this topic?**

Current research acknowledges that an autistic child can benefit from the presence of a certified canine. Such a bond is referred to as the child canine bond. Whilst there are many studies pertaining to animal assisted interventions there exists a dearth of research both national and international pertaining to the concept of animal assisted education.

**How does this research contribute to the field?**

This study contributes to research findings that are currently available. More specifically this study investigates:

• The child canine relationship within the autistic population.

• How a trained canine can impact the social development and cognitive functioning of an autistic child.

• Explore attitudes, experiences and perceptions of parents, canine handlers toward animal assisted education.

**What are research implications toward theory, practice, or policy?**

This study informs future policymakers and practitioners regarding animal assisted interventions and more specifically animal assisted education. An analysis of the data gathered in this study clearly acknowledges the fact that the contribution of parents, canine handlers and teachers is essential to the effective application of animal assisted education. Their opinions, attitudes and perceptions are necessary should the concept of animal assisted education be brought to fruition in the educational environment both nationally and internationally.

## Introduction

A broad range of research findings indicate that since the introduction of animal assisted interventions involving certified canines, the overall development of autistic children has been enhanced.^1^ However, there is no Irish policy or educational documentation that refers to animal assisted interventions as an effective intervention program for autistic children. This article reports on a recent study that examined the emerging phenomenon regarding the use of autism assistance canines to support the development of autistic children in Ireland. This qualitative study aimed to add to the body of research that already exists, with a view to informing future policy making both in Ireland and internationally.

Whilst the concept of autism assistance canines for autistic children has become an increasingly popular topic of research worldwide,^2,3^ it is clear that additional research pertaining to the benefits of autism assistance canines needs to be conducted. Furthermore, an evaluation and analysis of national educational policies and literature revealed the fact that no research has been conducted in the concept of animal assisted education in Irish society. Therefore, this paper supplements and enhances the current level of research in an endeavor to address the disparity in literature and investigate how the life experience of an autistic child is influenced by the bond established with an autism assistance canine. In this study the focus on parental, canine handlers and teachers’ perspectives provides an insight into the benefits associated with animal assisted interventions and autistic children within Irish society. This study explores the following research question:

-  How can Autism Assistance Canines influence the life experience of an autistic child from a parent, canine handler and teacher perspective in Irish society?

The research question was divided into a total of 3 embedded questions that are interrelated and correlate to the primary research question:

-  How can an autism assistance canine support the social skill development of an autistic child?
-  How can an autism assistance canine impact family functioning?
-  How does an animal assisted intervention involving a canine assist the learning experience of an autistic child?

## Literature Review

Current research endorses and supports the fact that the majority of autistic children demonstrate attachment behaviors.^4^ Attachment behaviors can be defined as close relationships with humans or animals.^4^ Other researchers suggest that autistic children are more likely to develop close relationships with animals as opposed to humans.^1,5^ An increasing number of studies have been published concerning the role a canine can have on the life experience of an autistic child.^4,6,7^ Research findings clearly indicate that the child canine bond can benefit the overall development of an autistic child.^8^ Ávila-Álvares^1^ more specifically discovered that this attachment bond is even more apparent in younger autistic children. To date, research published clearly demonstrates the necessity for further research and development into individualized therapies for autistic people, including animal assisted interventions.

Animal assisted interventions are considered an alternative intervention for autistic children.^9^ An increasing number of studies have been published concerning the role a canine can have on the life experience of an autistic child.^6,7^ Numerous researchers have argued that autistic children are capable of developing close relationships with animals, particularly with canines.^5,6^ Despite many definitions for animal assisted interventions, the most comprehensive definition, published by the International Association of Human Animal Interaction Organisation^10^ white paper, defines animal assisted interventions as structured goal-oriented interventions that intentionally incorporate animals in education, health and human services for the purpose of therapeutic gains in humans. On the basis of this definition, O’Haire^9^ advocate the inclusion of autism assistance canines under the category of animal assisted intervention. However, Brelsford^11^ caution that animal assisted intervention is a relatively new research field and criticize the lack of standardized guidelines available and universal ethical guidelines relating to human animal interactions in an educational setting. Whilst there are 3 forms of animal assisted intervention (a) animal assisted therapy, (b) animal assisted education, and (c) animal assisted activities, Fine^12^ suggests that scientific evidence confirms that all 3 forms of animal assisted intervention are equally respected when teaching autistic children.

A certified canine can improve the social and communication skill development of an autistic child, particularly young children.^1^ More specifically, research has concluded that a certified canine can increase positive behaviors, verbal communication and eye contact.^5,6,13^ Research suggests that a certified canine is responsible for an enhancement in social initiations and a diminishing level of social prompting of an autistic child.^9,14,15^ In this research study, we incorporate these research findings and include the perspectives of parents of autistic children in an endeavor to comprehend the unique bond established between a certified canine and an autistic child and the social benefits.

A reduction in the problematic behavior of an autistic child when in the presence of a certified canine has been clearly documented by Berry et al.^6^ The reduction in problematic behavior is attributed to the tactile interaction directly associated with the human animal bond.^6^ Many researchers have documented the multi-sensory stimulation associated to interaction with a canine reduces behavioral issues in an autistic child.^2,16,17^ Furthermore, scientific evidence supports the correlation between child canine bond and diminishing levels of stress.^18^

The functioning of a family unit can be enhanced by the presence of a certified canine.^6^ Other researchers, including Burgoyne et al^19^ and Harwood et al^4^ support this assertion and suggest that not only does a certified canine improve family functioning but also inspires family interaction. Furthermore, Burrows et al^17^ noted that the presence of a certified canine enhances family activity including social outings, whilst diminishing embarrassment and stress levels when in the public eye. Similarly, Sprod and Norwood^2^ identified the positive influence associated with wearing of identifiable apparel on autism assistance canines in public. In contrast, Burgoyne et al^19^ referred to challenges associated with the ownership of a certified canine. Such challenges included financial implications, emotional attachment between child and canine and mortality and long waiting lists for autism assistance canines in Irish society. While much of the research literature supports the enhancement of family life through the presence of a certified canine, this particular area of research is in its infancy and therefore additional research is necessary.^20^

The application of animal assisted education in an education setting is beneficial for autistic children.^11,16^ Many researchers have documented these benefits. Such benefits include an enhancement in social skill development, positive social behaviors, reduction in problematic behavior, diminishing levels of stress, potential to increase a child’s literacy attainment and increase motivation.^8,9,21,22^ However, a noticeable limitation of research conducted to date is the absence of participation by parents, teachers and canine handlers.^21^

Animal assisted education is only occasionally applied in classroom settings worldwide.^5^ According to Hill et al,^5^ this is because school principals are uncertain whether or not they can permit the use of service animals in public schools. In Ireland, the lack of guidelines and standards regarding animal assisted education is apparent within the Irish education system and whilst a number of Irish health and educational documents make reference to supports available for autistic children, not one Irish document refers to animal assisted education. Despite the mass volumes of policies available, it is unfortunate the concept of animal assisted education has neither been included nor referenced. To fulfill an all-inclusive educational system, additional research in the concept of animal assisted education is necessary. Furthermore, according to Smith and Dale,^23^ the more formal animal-based interventions in classroom need to be adequately designed and evaluated.

## Method

A qualitative research design was used to address the research question through the exploration of 9 participants’ real-life experiences concentrating on specific verbal data rather than statistical data accrued.^24^ The study used narrative inquiry to gather a number of participants’ stories and individual experiences using semi structured interviews.^25^ This approach facilitated the merging of multiple viewpoints.^25^ Nine semi structured interviews were conducted over the telephone. This facilitated the sharing, recording of individual stories and experiences whilst facilitating the geographic distribution of the participants.^26^ Furthermore, due to the COVID-19 global restrictions telephone interviews were deemed most suitable for this study. Interviews were recorded using a Dictaphone. Data gathered were transcribed, reviewed and analysed. Furthermore, a debriefing period at the end of every interview provided the participant with an opportunity to include additional information. The duration of interviews was 45 to 60 min.

The interview questions were informed by the embedded research questions. The majority of the interview questions were open ended and underpinned by literature to ensure rigor.^26-28^ Sample interview questions for parents include: *How would you describe the relationship between your child and your canine? Do you think the autism canine has made a difference to your child’s social skill development? What are the challenges of an autism assistance canine?* Sample canine handler interview questions include: *Can you describe the autism assistance canine training process? What are the benefits of an autism assistance canine in your opinion?* Sample Teacher interview questions include: *During your teaching career have you experienced the presence of a certified canine in your classroom? What are the challenges associated with an autism assistance canine in the school environment? Have you completed any CPD courses relating to animal assisted intervention?*

### Sampling Approach

The study adopted a bilateral sampling approach that utilized the concept of purposive and snowball sampling. Purposive sampling was initially introduced whilst snowball sampling was subsequently introduced. Purposeful sampling facilitated the identification of a population that provided accurate information whilst snowball sampling enabled the researcher to identify cases of interest from people who are familiar with other people referred to as information rich cases.^27^ The sampling population was formed on 3 particular cohorts identified as parents, canine handlers and teachers. This small sample size was justified by Yardley^29^ who claimed that rigor is not determined by the sample size but by the information provided by participants. This study comprised of 4 parents, 3 canine handlers and 2 teachers. All parent participants in this study are parents of autistic students. Additionally, all canine handler participants had at least 3 years’ experience and worked closely with autistic students and respective families. Similarly, all teacher participants had 3 years or more teaching experience and taught numerous autistic students. Participation of all 3 cohorts was deemed to be essential to determine a well-balanced research study that contributed to the dearth of research available.

### Research Integrity

Planning and conducting an ethical study reduces bias and minimizes risk to potential participants.^27^ In the present study, ethical consent acknowledged that participation in this study was voluntary, and all participants had the right to withdraw from the study at any time without consequence.^30^ Additionally, the researcher guaranteed participant confidentiality and anonymity throughout the study by providing each participant with a random ID number to ensure non-traceability. All data published were accurate.

### Data Analysis

Braun and Clarke’s^31^ 6-step thematic approach was used to analyze data. Data were transcribed from audio transcripts using an orthographic style, data similarities and dissimilarities noted, data was coded and thematic analysis utilized to identify identifying key themes. A concise, comprehensible and noteworthy account of the story the data communicated was identified.

### Reliability and Validity

As mentioned, data were analyzed in accordance with the Braun and Clarke’s^31^ 6-phase approach to data analysis. This approach entailed the generation of codes from the data accrued, the searching and reviewing of data for themes and finally, defining and interpreting the themes. Furthermore, the initial validation strategy entailed validity from the researcher’s lens and involved the use of a reflective journal. A number of categories were observed and recorded in the research journal including participant engagement, researcher views, opinions and self-reflexivity.^32^ In addition to the reflective journal, member checking and validity from a readers’ lens were utilized to ensure reliability and validity.

## Findings and Results

The findings generated from the 9 semi structured interviews are thematically presented. The 4 primary themes that emerged from the data analysis are: (a) child canine bond, (b) social development, (c) family, and (d) animal assisted education.

### Child Canine Bond

All 3 canine handler participants in this study said that attachment walks were a vital component of the canine selection process. Attachments walk is defined as a Similarly, all 4 parent participants in this study attributed the bond between their child and canine to the comprehensive canine selection process and attachment walks. One parent claimed that: *it’s like a step by step process. . .we got a match . . . the bond is definitely there.*

During the course of this study differing opinions of both parent and canine handler participants became evident. Two of the 3 canine handler participants acknowledged that the bond established between an autistic child and a certified canine was not always guaranteed. “the relationship is just not guaranteed . . . sometimes with the child does not like dogs or something like that.” In comparison, an analysis of data found that an assumption of parents was that the child will bond instantaneously with the canine. *I suppose we initially just assume that the bond will be instant, and we also assume that you know they will bond and skip off into the sunset being attached*.

### Social Development

The analysis of data acquired from parents and canine handlers endorsed the suggestion that the social interaction, safety, independent functioning, companionship and improved language development of an autistic child was owing to the presence of an autism assistance canine.

Parents attributed an increase in social initiations to their child’s enhanced safety and improved behavior and attributed this improvement to the allocation of a certified canine. In this study, the connection between safety and flight risk of an autistic child was articulated. Two of the parent participants suggested that the primary purpose for availing of an autism assistance canine was to enhance the safety of their child. The introduction of an attachment belt was directly attributed to the enhancement of the safety of an autistic child. Both canine handlers and parents referenced this stating that an autism assistance canine can positively influence the safety of an autistic child outside of the home environment. Furthermore, enhanced social communication owing to increased safety was also referenced by parents in this study, more specifically the social skill of eye contact. One parent claimed that her child “has more eye contact.” Alongside this skill, the social skill of smiling was referred to by another parent participant. Parent participants in this study were of the belief that such social skill development can be directly attributed to the allocation of an autism assistance canine.

The data from this study also suggested that the presence of an autism assistance canine contributed to a noticeable improvement in independent functioning. Two of the parent participants acknowledged the enhancement of their child’s independence with the introduction of an attachment belt. One parent stated that their child was *definitely more independent walking and stays by the dogs side . . . they don’t budge from each other.*

The concept of companionship was introduced by both parent and canine handler participants. A parent participant suggested that the benefit from the allocation of an autism assistance canine to her child was friendship and described the canine as a “forever friend.” Similarly, a canine handler participant referred to the canine as a “buddy” for a child with ASD. Furthermore, from the observations disclosed by parent participants, it could be construed that strangers are more likely to engage with an autistic child whilst the child is accompanied by a certified canine.

Additionally, canine handlers introduced the concept of language development for both verbal and minimally verbal autistic child. One canine handler recalled details of her experience with an autistic child that she has previously worked with. The canine handler recalled details of this experience as follows: He had some words until he was 3 and then he lost them, and I trained him with his dog and within 4 months his first word was his dog’s name and a couple of months later. . . he eventually got up to 5 words. The findings of this study suggested that the presence of a certified autism assistance canine may also be beneficial to minimally verbal autistic children. This finding supports the contention that the presence of an autism assistance canine can contribute to the enhancement of language development.^1^ Notably, all parent participants in this research study claimed that the presence of a certified canine could be directly attributed to the development of their child’s language. One of the canine handler participants together with one of the parent participants also referred to this concept in this research study. However, one canine handler emphasized that language development is “not something we promote, it is not always guaranteed but it does happen.”

### Family

Both parent and canine handler participants, suggested that an autism assistance canine can impact families of autistic children. Parent participants indicated that with the evolvement of time the certified canine integrated with family members and became part of the family. Parent participants described the canine as a family member, one parent claimed their certified canine was “a seventh child.”

Inclusivity of the family unit is the most significant development associated to certified canine ownership. A canine handler acknowledged that family members benefit as the certified canine for the parents “is a peace of mind they can go out with the child.” All parent participants reported an increase in family outings which are attributed to the arrival of an autism assistance canine. Furthermore, 2 of the parent participants expressed the belief that the bond between their autistic child and siblings had noticeably increased since the arrival their respective certified canines.

Significant high levels of stress are correlated to the challenges concerning parenting autistic children.^19^ Autism assistance canines have been attributed to a noticeable reduction in parental stress levels and are reiterated in the findings of this study.^2,19^ All parent participants acknowledged the level of stress associated with parenting children with ASD. Furthermore, the parents in this study clearly acknowledged a reduction in the levels of stress since the arrival of an autism assistance canine. One parent stated that their autism assistance canine “was like a therapy dog for me.” Additionally, the therapeutic benefits associated with the ownership of a certified canine were described by one of the parent participants. One parent stated that “I rub him and everything will be alright.” It is evident from the findings that the presence of an autism assistance canine provides significant support to both the child and extended members of the family. Similarly, 1 parent participant suggested that the presence of their certified canine during homework activities provided a calming influence on her son. Furthermore, this participant believed that the interaction between her child and the certified canine assisted in her child’s ability to focus on the task at hand.

All parent participants in this research study attributed tactile interaction to the reduction in their child’s problematic behavior. This is in keeping with Berry et al’s^6^ assertion that a reduction in problematic behavior is owing to the tactile interaction provided by a canine. In this study, a reduction in problematic behavior had been noted both within the home environment and whilst in public. Parent participants suggested that their children would “cuddle” or often be “wrapped around” the canine. Findings by Beetz^16^ harmonize this finding suggesting that the multisensory stimulation associated with canine interaction reduces behavioral issues in autistic children. It is apparent from the qualitative data gathered throughout this study that tactile interaction provided by a canine is associated to a reduction in problematic behavior in autistic children.

The challenges associated with certified canine ownership were referenced by all participants in this research study. In particular, time needed to facilitate maintenance and exercise has been identified by the participants. One parent participant emphasized that the “dog requires a maintenance walk as well as an attachment walk.” Additionally, supervision encapsulates the challenge of time and is an essential aspect of safety. The owner of a certified canine has to be cognizant of the issue of supervision at all times. However, from the canine handler’s perspective, the issue of supervision is not as challenging. Furthermore, similar to the findings of Burgoyne’s study, both parent and canine handler participants identified the **l**ong waiting lists as an additional challenge for parents of autistic children in Ireland. More specifically one canine handler participant stated in in September we had 200 people apply for the waiting lists and we could only take about 70. There can be no doubt that challenges arise with ownership of a certified canine. Canine handlers emphasized the importance of parents of autistic children being fully conversant with the challenges associated to ownership of an autism assistance canine. One canine handler stated that parents should be made aware of these challenges well in advance of ownership of a certified canine. Therefore, there can be no doubt that parents feel better equipped to deal with their child’s needs with the presence of an autism assistance canine.

### Animal Assisted Education

The findings of this research study suggested that Irish education policies have not considered the concept of autistic children being accompanied to school by a certified canine. This research study suggested that future educational policies should afford consideration to individual cases dependent on circumstances and individual needs. The data gathered from parent participants conveyed that the presence of an autism assistance canine in the school environment could be beneficial to an autistic child. Participants of this research study have referenced this issue and have strongly advocated the benefits such a practice would accrue. From the participation of the canine handlers in this research study it has become apparent that the training process does not incorporate integration of autism assistance canines into the school environment. The current canine training program neither includes nor recommends participation by individual teachers. Should the concept of the integration of autism assistance canines into the school environment be advanced, inclusion and participation of teachers would be essential. A degree of responsibility would automatically transfer to the teacher and therefore, a certain level of training would be required. Teacher participants in this research study have not referenced animal assisted education in school policies. As a consequence of the absence of any Irish education policy pertaining to animal assisted education, no continuing professional development courses are available to teachers in this particular area. This finding was referenced by both teacher participants in this study when they acknowledged the benefits of canine accompaniment for an autistic child within the school environment. An analysis of the data suggest that such benefits outweigh the challenges.

## Discussion

Analysis of the data collated suggested that an autism assistance canine can benefit the safety, social interaction, independent functioning, companionship and language development of an autistic child. In particular, the findings suggested that an autism assistance canine can positively influence the behavior and family life of an autistic child. The application of animal assisted intervention involving autism assistance canines in Irish primary schools is challenging. These challenges arise due to the absence of government and education policies in the area of animal assisted education.^13^ A comprehensive review of these policies is necessary in order to advance this issue.

The findings of this research study strongly suggested that an autism assistance canine can positively enhance the life experience of an autistic child. Analysis of the data collected from the participants, including parents, canine handlers and teachers, clearly supported this finding. Central to this study were 3 embedded questions a summary of which is outlined below.

### How Can an Autism Assistance Canine Support the Social Skill Development of a Child With Autism Spectrum Disorder?

Grigore and Rusu^14^ emphasized that a canine can enhance the social skill development of an autistic child. The research findings from this study supported this finding. Both parent and canine handler participants said that an autism assistance canine facilitated social interaction for autistic children. Similar to findings of Berry et al,^6^ Hill et al,^5^ and O’Haire,^13^ this research study reported an increase in positive social behaviors and verbal communication. The improvement in social skills was attributed to the enhanced safety of the child, a finding posited by O’Haire et al.^9^ Teacher participants expressed the opinion that an autism assistance canine could positively impact the social skill development of an autistic child. Furthermore, they acknowledged the dearth of educational policy in this particular area.

### How Can an Autism Assistance Canine Impact Family Functioning?

The positive influence an autism assistance canine has on family life became apparent from an analysis of the qualitative data gathered. Inclusion of the certified canine into the family unit became evident and the consequence was an enhanced bond between family members. Inclusivity of the entire family unit was considered the greatest benefit since availing of an autism assistance canine.

Parents and canine handlers highlighted the increased safety associated with canine ownership. Consequently, families noticed a greater level of freedom that facilitated more frequent outings. This increased activity was directly attributed by parents to a significant reduction in parental stress levels. These findings are supported by the research of Burrows et al,^17^ Harwood et al,^4^ and Wright et al.^20^

Challenges associated with canine ownership were referenced by both parents and canine handlers. However, when these challenges were considered in association with the benefits accrued, it immediately became evident that such benefits clearly outweighed the challenges. This finding of the research study is in accordance with the findings of a study conducted by Burrow et al.^17^

### How Does an Animal Assisted Intervention Involving a Canine Assist the Learning Experience of a Child With Autism Spectrum Disorder?

Despite the fact that there is no reference to animal assisted education within the Irish education policies, teacher participants expressed knowledge of and an interest in this area of education. One teacher had outlined the benefit of a certified canine in the school environment. The benefit of animal assisted education to the education of an autistic child has been endorsed by all participants of this study. The difficulty associated with the implementation of animal assisted education has been acknowledged and is directly attributed to the absence of policy within the Irish educational system, a finding that has been clearly evident from this research study. The absence of policy is not unique to Ireland and this can be seen in the findings of a study by Hill et al.^33^ Hill’s study identified that there was a level of uncertainty regarding the application and implementation of animal assisted education within educational settings by school managers worldwide.

## Recommendations

This study has incorporated the experience and opinions of parents, canine handlers and teachers alike. An aim of this study was to provide research information that may be of assistance to future policy makers in considering the educational needs of autistic children. The findings of this study supported the findings of a study conducted by Ávila-Álvares et al^1^ and suggested that the presence of an autism assistance canine can positively enhance the safety, social development and social interaction of an autistic child. Furthermore, the findings of this study suggested that incorporating certified canines into the education of autistic children would enhance independent functioning. Therefore, the need to design and implement educational policies that incorporate animal assisted intervention would enhance the Irish educational system by fostering an inclusive educational environment.^34^

The findings of this study clearly suggested that it is incumbent on future educational policy makers to increase awareness of animal assisted education in educational settings in advance of the implementation of any practice. An analysis of the data gathered from this research study suggested that a multilateral approach needs to be considered to incorporate effective communication and training for all interested parties. Additionally, all canine handler participants in this study emphasized the need for an effective teacher and parent support program to be designed to incorporate the opinions and expertise of recognized canine organizations. The suitability of participants due to the individualistic nature of autism spectrum disorder, as referenced by Smith and Dale^23^ and acknowledged by participants in this study, would have to be afforded consideration. Finally, schools could engage in sensory and environmental audits which consider the role of canine assistance in the school.

The dearth of international studies pertaining to the concept of animal assisted education and the absence of national studies in this area of education is clearly evident. This qualitative study surveyed the perceptions and attitudes toward the concept of animal assisted education within the Irish educational system from the perspective of parents and teachers of autistic children and canine handlers. It was apparent from the data analyzed that additional and more comprehensive studies are required in order to provide an accurate assessment of the benefits associated with animal assisted education. A research study with a larger number of participants would provide more comprehensive and accurate findings. Additionally, a study considering the development of an auditing tool for the inclusion of canine assistance in schools is required.

## Limitations

It is unfortunate that the study was conducted during the current global pandemic, the pandemic of COVID-19. Restrictions in personal contact, direct communication, travel and school closures were introduced into law and implemented by the Irish government. Such restrictions meant that there was no opportunity to conduct interviews in person. Observation of the interviewee would have afforded the opportunity to enhance this study by adding to its validity.^25^

Creswell^25^ suggested that a small sample size can be a limitation, however, in this study the small sample size provided an in-depth understanding of the topic being explored. A sample size (*n* = 9) for this study provided an insight into the participants perspectives however, a larger sample size may have yielded conflicting results. In this study snowball sampling enabled the researcher to identify cases of interest from people who are familiar with other people referred to as information rich cases.^25^ However, Creswell^25^ clearly acknowledges that snowball sampling can distort the outcome of a research study.

## Conclusion

This article focused on the benefits an autistic child can gain from the presence of an autism assistance canine. There is compelling evidence available in research literature published, reiterated in this qualitative research study, to support the critical role an assistance canine can play in the safety, behavior, social skills and education of an autistic child.^6,13,16,17,19^

An analysis of the data gathered in this study clearly acknowledges the fact that the contribution of parents, canine handlers and teachers is essential to the effective implementation of animal assisted education. Their opinions, attitudes and perceptions are necessary should the concept of animal assisted education be brought to fruition in the educational environment both nationally and internationally. Despite the lack of government policies in Ireland, positive attitudes and perceptions toward animal assisted education are clearly evident. Undoubtably, further research regarding the implementation of animal assisted education in an educational environment is required. The recommendations made from this research study should be considered for the Irish educational system in order to embrace a more inclusive system. Such inclusivity would clearly enhance the Irish educational system. This qualitative study supports existing research findings that an autism assistance canine can benefit the safety, social interaction, independent functioning, companionship, language development and the educational experience of an autistic child.
